# Hæmatology.—IV

**Published:** 1910-05-07

**Authors:** 


### PATHOLOGY.

### HÆMATOLOGY.—IV.

3. The Spectroscopic Examination of the Blood.

This is seldom employed, but the method is of special value in incipient blackwater fever cases where it may be necessary to determine if methæmoglobin is present in the serum before it appears in the urine. A fairly large quantity of blood is required for the test, and this may be obtained by a cupping glass. After drawing the blood, dilute it with water till it becomes of a cherry-red colour. This is placed in a thin flat glass tube, or in a small glass vessel, with two sides inclined towards each other, so that various thicknesses of the fluid may be examined. Different dilutions of the blood may also be made. A hand spectroscope is usually employed, but the disadvantage of any of these forms is that it is by no means easy to see or read the bands on the spectroscope. Oxyhæmoglobin, reduced hæmoglobin, carbonic oxide hæmoglobin and methæmoglobin can all be detected by their characteristic absorption bands, diagrams of which are given in every good text-book on physiology.

4. The Determination of the Coagulation Time of the Blood.

Wright's tubes are used for determining the coagulation time of the blood. A series of fine tubes of equal calibre are kept at a uniform temperature by being placed in a water jacket. Blood is then drawn into each of these at definite intervals of time. At intervals of half a minute blow down the tubes, the interval between the filling of the tube and the occurrence of coagulation is known as the coagulation time. Coagulation is diminished in hæmophilia, purpura hæmorrhagica, and some other diseases.

5. The Estimation of the Specific Gravity of the Blood.

There is a relation between the hæmoglobin and specific gravity of the blood, a low hæmoglobin indicating a low specific gravity. If one has estimated the former, therefore, there is no special necessity for also doing the latter; but still the method may be described, as it may prove useful if one has no hæmoglobinometer by one. Hammerschlag's method is the best and simplest. It depends on the fact that chloroform is heavier than blood, benzol or turpentine lighter. Mix these two fluids (chloroform and benzol or chloroform and turpentine) in a urine glass in such a proportion that the specific gravity of the mixture is 1059—*i.e.* that of normal blood. Suck up a little blood in a pipette and blow drops out from this into the mixture. These float like little red beads, and may either sink to the bottom, rise to the top, or remain in the middle. If they sink add chloroform, if they float add benzol, and go on adding either of these till the bead remains stationary in the middle. When it does this take the specific gravity of the mixture, and this gives the specific gravity of the drop of blood. Cabot gives the following precautions for doing the test: (1) Have the inside of the urinometer quite clean and dry; (2) have more than one drop of blood; (3) add the re-agents drop by drop, and stir; (4) avoid having any air in blood-drop; (5) do as quickly as possible, otherwise the chloroform or benzol may work into the blood-drop and affect its weight. The importance of the method is that by using a table (see Cabot's book) the hæmoglobin may be calculated; a low hæmoglobin, as we have just said, indicating a low specific gravity. (Example: 1033—1035=HO. 25 to 30 per cent.; 1057—1060=HO. 85 to 95 per cent.) The chloroform-benzol mixture can be filtered, kept in a bottle, and used again. The method is useless for dropsical cases, and is also inaccurate in pernicious anæmia and leukæmia cases.

6. The Agglutination Reaction.

This is more popularly known as the Widal reaction, and is of great value in the diagnosis of at least two diseases—typhoid and Malta fever. If a person is suffering from (after the seventh day) or has had typhoid fever, then his blood serum acquires what has been called an agglutinative action to the specific organism (the typhoid bacillus), causing these, when suitably prepared, to stop moving and to collect into little clumps or masses; dead bacilli are also acted upon in the same way. The list may be carried out in two ways—*e.g.*, micro- or macro-scopically. The former of these is strictly the agglutination reaction, this meaning the collection into clumps and loss of motility (where living cultures are being used) of well-spread-out bacilli in a fluid medium; the latter is the sedimentation test, this meaning the deposit formed of such a series of clumps when the fluid is allowed to stand. Agglutination is carried out on a slide and examined under a microscope; sedimentation in upright glass tubes and watched with the naked eye. The serum is too potent to use undiluted, so it must be diluted before use, two or three different strengths usually being employed. *To do the reaction for typhoid.*—Draw blood from the suspected case in the usual manner. Fill capillary tubes with this and centrifuge these quickly to separate the serum. Next take a young typhoid culture (18 to 20 hours old), either in broth or on agar; if on the latter, scrape growth off with a platinum needle and make an emulsion in broth or distilled water. Filter culture through filter paper, taking a drop of the result and making a hanging drop preparation so as to test the activity and general condition of the bacillus (first control). If this is good, and the germs are evenly distributed and active, then the test may be proceeded with. A second control is sometimes used—namely, a diluted normal serum (say, one's own serum) being added to the bacilli; with this, of course, no clumping should take place. Two different dilutions (1-25, 1-50, are useful figures) of the suspected serum are then added to the bacilli and the result noted. If the reaction is to take place, the bacilli will begin to adhere to one another, will lose their motility, form clumps, and, after the lapse of some time, practically no free ones will be seen moving about; if, on the other hand, it is not to take place, then the bacilli will go on moving about and will not form proper clumps within the given time limit. If a few small clumps form, but most of the bacilli go on moving about actively, then the reaction is not conclusive, and the result should be returned as negative. It is only by being very strict with the interpretation of one's results that one is uniformly successful, and if two dilutions —1-25, 1-50—are taken, with a time limit of half an hour, and only complete and proper clumping is given as a positive result, then one should make few mistakes. All that one is required to say (at least in the laboratory) is whether the reaction is positive or negative, and then it is left with the clinician to decide whether or not the case is one of typhoid.

